# Effects of pre-transplant azithromycin administration on kidney graft function: study protocol for a double-blind randomized clinical trial

**DOI:** 10.1186/s13063-018-2744-y

**Published:** 2018-06-28

**Authors:** Gholamreza Mokhtari, Mojtaba Teimoori

**Affiliations:** 1 Transplant surgeon, Urology Research Center, Razi Hospital, Guilan University of Medical Sciences, Rasht, Guilan Iran; 2 Urology Department, Guilan University of Medical Sciences, Rasht, Guilan Iran; 3 Urology Research Center, Razi Hospital, Sardar jangle St, Rasht, Guilan Iran

**Keywords:** Azithromycin, Kidney transplantation, Immunomodulation, Graft rejection, Transplant recipients

## Abstract

**Background:**

Kidney transplantation is the best strategy for the management of end-stage renal disease; however, the outcomes need to improve further. Macrolides show antimicrobial and anti-inflammatory properties in chronic diseases and intraoperatively, and can accumulate in tissues for extended periods. Therefore, theoretically, when administered to a donor and because of accumulation in the donor kidney, macrolides can cause graft immunomodulation and improve kidney transplantation outcomes.

**Methods:**

This study is a single-center, randomized clinical trial. A total of 60 kidney donors will be randomly allocated to the azithromycin or placebo group and treated with a single dose (1 g) of azithromycin or placebo, respectively, 1 day before surgery. Recruitment commenced in September 2016 and is expected to be completed by March 2018. The primary outcome is kidney graft function. The secondary outcomes include rejection rate, urinary tract infections in graft recipients, pain and systemic inflammatory response syndrome in live donors, and complications in both donors and recipients. Outcomes will be evaluated at baseline and every day in the first week after transplantation, as well as at 1 and 3 months post transplantation. Adverse reactions will be documented. If the efficacy of azithromycin in reducing adverse outcomes is confirmed, it would serve as an easy to use, economic intervention able to lower post-transplantation risks.

**Discussion:**

Short and mid-term analyses of blood and urine samples as well as immunological assays will facilitate a more in-depth analysis of the effects of azithromycin on transplantation outcomes.

**Trial registration:**

Iranian Clinical Trial Registry, IRCT201606141853N11, registered on September 5, 2016.

**Electronic supplementary material:**

The online version of this article (10.1186/s13063-018-2744-y) contains supplementary material, which is available to authorized users.

## Background

The incidence and prevalence of end-stage renal disease necessitating dialysis or transplantation has not changed in the past few decades, with these even increasing in developing countries, wherein healthcare needs to be improved in line with the burden of disease [1, 2]. Compared to dialysis, a kidney transplant affords significantly better survival and quality of life to end-stage renal disease patients, making transplantation one of the most cost-effective surgeries [3]. Therefore, preventing graft loss after kidney transplantation is crucial.

The immunomodulatory properties of antibiotics such as macrolides have been studied and reported [4–6]. Azithromycin is a member of the macrolide (azalide) family and its blood serum concentration can be ≥ 100-fold that in body tissues, especially in leukocytes [7, 8]. It can accumulate in body tissues for up to a month even following administration of a single dose [9]. Therefore, azithromycin administered to kidney donors may persist in transplanted kidney cells, especially in the immune cells of the grafted kidney.

Mammalian target of rapamycin (mTOR) is a cytoplasmic protein that plays a key role in the activation of immune cells following foreign stimulation and is now believed to be a major immune system controller [10], making rapamycin one of the most important drugs in transplantation [11]. Ratzinger et al. [11] showed that azithromycin exerts an immunomodulatory effect on CD4 (+) T-cells by preventing mTOR activity. Thus, administration of azithromycin prior to a transplant can result in prolonged drug accumulation in the kidney and modulate inflammatory processes locally.

The immunomodulatory influence of macrolides has been shown to lower the expression of inflammatory genes [12] and affect the production and efficacy of many pro-inflammatory cytokines such as interleukin (IL)-1, IL-6, IL-8 and tumor necrosis factor [13, 14]. Chemoattractant and inflammatory molecules have been reported to be crucial determinants in kidney rejection [15, 16]. Azithromycin can limit the signaling of chemoattractants such as IL-8 and reduce the production of inflammatory molecules such as granulocyte macrophage colony-stimulating factor, which contribute to neutrophil attraction to inflamed organs. Thus, the anti-inflammatory action of macrolides can prevent leukocyte infiltration into the transplanted kidney.

To our knowledge, no published study has evaluated the effects of macrolide administration in kidney donors on outcomes in kidney recipients. Azithromycin administration to cadaveric or live donors can theoretically result in drug accumulation in the donated kidney, where it exerts an immunomodulatory effect and can reduce preoperative adverse outcomes such as rejection. The effects of azithromycin in other organ transplantations have been previously shown. Azithromycin therapy following keratoplasty yielded results similar to those achieved with topical dexamethasone, without the risk of adverse effects commonly associated with steroidal drugs and enhanced graft survival in rats [17]. A study found that azithromycin promoted the survival of high-risk corneal allografts, apparently without modulating the immune system [18]. Iwamoto et al. [19] reported that azithromycin, with its immunomodulatory properties, is a potential prophylactic drug for the lethal graft-versus-host disease.

In this study, we will assess the safety and efficacy of azithromycin administration to kidney donors and its effects on both donor and recipient outcomes.

## Methods

### Overview of the study design

We designed a double-blind randomized clinical trial (RCT). The interventions will be performed at an academic institution, The Affiliated Hospital of the Guilan University of Medical Science, Guilan, Iran.

### RCT flow chart

Prior to inclusion, all participants will be screened at the Urology Research Center and will then be randomized into the azithromycin or placebo group. Following randomization, the participants will receive 1 g of azithromycin or placebo 1 day before surgery. The outcome will be evaluated by investigators blinded to the group randomization, during the study. Adverse events will be documented for safety assessment.

**Fig. 1:**
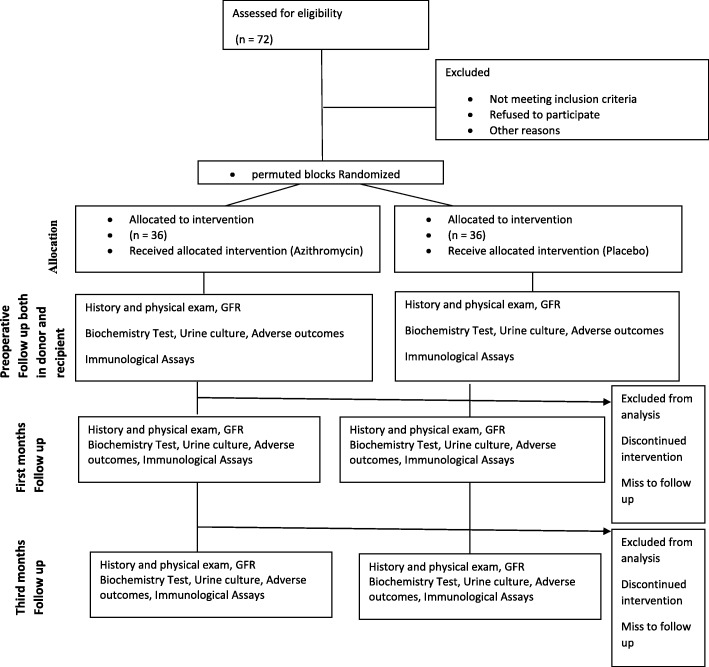
Consolidated Standards of Reporting Trials (CONSORT) diagram showing the flow of participants through each stage of a randomized trial

**Fig. 2:**
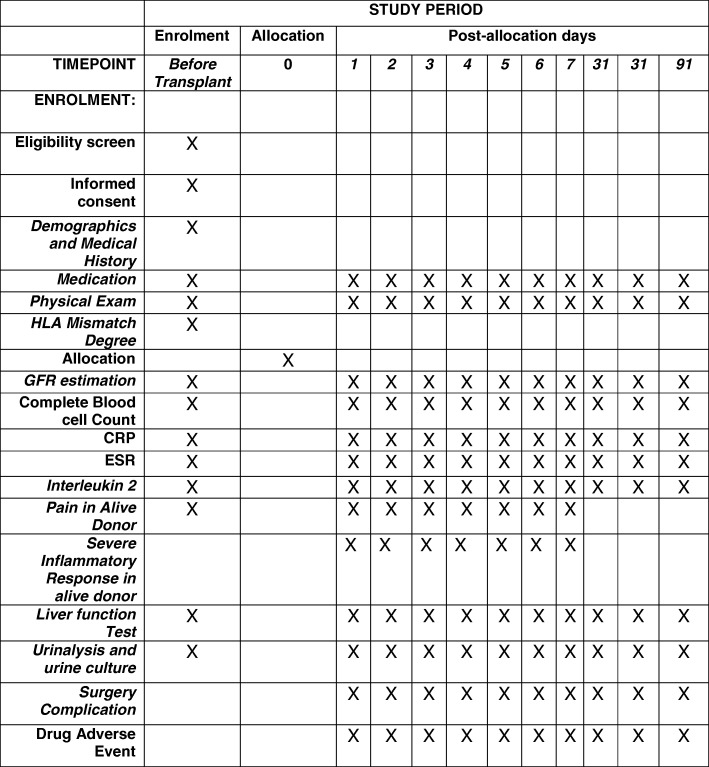
Standard Protocol Items: Recommendations for Interventional Trials (SPIRIT)

The study protocol has been reviewed and approved by local institutional review boards and ethics committees. The trial adheres to the principles of the CONSORT (Fig. 1), the Standard Protocol Items: Recommendations for Interventional Trials (SPIRIT) (Fig. 2) (checklist as an Additional file 1), and the Declaration of Helsinki (sixth revision, 2008).

### Participants

All procedures will be performed at Razi Hospital University Medical Center. All kidney donors considered for randomization would be informed of the study protocol. The standard preoperative screening of donors will include an examination by a nephrologist, a transplant surgeon, and an anesthetist. Renal ultrasonography, magnetic resonance angiography, or digital subtraction angiography will be performed to evaluate the vascular anatomy of the kidneys. If both kidneys are suitable for transplantation, the right kidney would be preferred. All recipients would undergo follow-up examinations every day for a week after surgery, and at 1 and 3 months post transplantation.

### Sample size

The sample size was determined based on the fact that 36 subjects per group would appear adequate to provide estimates precise enough to meet our aim (pilot study) [20].

### Inclusion criteria

1. Live or cadaveric donor.
2. Informed consent form signed by the live donor or consent provided by the guardian or next-of-kin for a deceased donor.

### Exclusion criteria

1. Known allergies, including allergies caused by azithromycin.
2. Severe heart, liver, or renal dysfunction or hematological, respiratory, cardiovascular, psychiatric, or metabolic disease within a 6-week period before transplantation.
3. Use of hormonal or other medication, including macrolides.
4. Concurrent participation in other clinical trials.

### Randomization

Participants will be randomly assigned to either the azithromycin or placebo group by using the method of random permuted blocks [21].

### Blinding

This is a double-blind trial; therefore, both the patients and the clinical practitioners will be blinded at all stages of the RCT. Furthermore, to prevent potential biases, other researchers, including data collectors and statisticians, will also be blinded to the trial.

### Interventions

Live donors in the azithromycin group will receive 1 g of azithromycin (four capsules of 250 mg) orally 1 day prior to surgery. Capsules will be opened, and the powder will be mixed with 10–15 mL of sterile water and immediately administered via a gastric tube in the case of deceased donors. The tube will be pre-flushed with 30 mL of sterile water; after administration, the tube will be flushed again with 30 mL of sterile water and clamped. The participants in the control group will receive a placebo. To ensure compliance, all live donors will ingest the capsules in the presence of a physician [22]. Possible side effects due to the azithromycin treatment will be monitored; to our knowledge, there are no contraindications for administering azithromycin through enteral feeding tubes [23]. In cases of serious side effects, the trial will be ended and treated.

### Immunotherapy protocols

Maintenance immunosuppression therapy in most recipients will consist mainly of cyclosporine. The majority of patients will also receive mycophenolate mofetil and prednisolone, except for those who experience side effects. All medical regimens will be recorded during hospital visits.

### Patient follow-up

Patients will be followed for 3 months after treatment. Clinicians will examine the patients every day for a week after surgery, and again at 1 and 3 months.

### Outcome measures

#### Primary outcome

The primary outcome will be the kidney graft function (evaluated using the glomerular filtration rate).

#### Secondary outcomes

Other studies have reported that prophylaxis with macrolides can decrease systemic inflammatory response syndrome [24, 25]. Chow et al. [24] reported that clarithromycin therapy reduced febrile response, tachycardia, tachypnea, and the strength and length of postoperative pain, and suppressed an increase in monocyte counts, concluding that macrolide treatment can yield improved clinical outcomes. Therefore, the secondary outcomes in our RCT will be the rejection rate, pain and systemic inflammatory response syndrome in donors, and complications and inflammatory responses in both donors and recipients.

Theoretically, azithromycin accumulated in the grafted kidney can decrease the rate of urinary tract infections (UTIs) after transplantation, because of the antimicrobial effect of azalide drugs. Azithromycin has been shown to inhibit UTIs caused by *Pseudomonas aeruginosa*, which are mainly catheter-associated UTIs [26, 27]; therefore, another secondary outcome in the study will be the incidence of UTIs in recipients.

### Outcome evaluation schedule

Outcomes will be assessed at baseline and at 1 and 3 months post transplantation. The patients will undergo routine blood tests, liver function tests, and kidney function tests. The overview of the outcome evaluation schedule is shown in Fig. 1.

### Assessment of adverse events

According to previous RCTs, azithromycin administration may cause several types of adverse events, including hearing loss, cardiac arrhythmia, nausea, and vomiting [28]. Adverse events will be carefully recorded in case report forms.

### Statistical analysis

We will use standard descriptive statistics to assess baseline clinical and laboratory data at enrollment. Subsequently, we will compare creatinine levels at the end of each study period by using mixed models for repeated measures. Fixed factors will include the degree of human leukocyte antigen mismatch, age, sex, etiology of and baseline comorbidity, medication (azithromycin or placebo), and baseline laboratory test parameters (inflammatory and non-inflammatory markers). Complications, UTIs, and other outcomes will be similarly evaluated. Nominal data will be compared using the Student’s T test and categorical data will be compared using the χ^2^ test. Patients who drop out during the study period will be analyzed until the last hospital visit at which data are collected. *P* < 0.05 will be considered as a significant level. All data will be analyzed using SPSS software Version 20.

## Discussion

The strength of this trial is that it is a well-designed, novel, double-blind RCT. It will provide evidence of the effect of azithromycin on kidney transplantation outcomes. The secondary objectives will allow us to examine the effect of azithromycin on UTI rates in recipients and pain and inflammatory responses in both donor and recipients. Further, short and mid-term analyses of blood and urine samples as well as immunological assays will enable a more in-depth analysis of the effects of azithromycin on transplantation outcomes. On the other hand, the limitation of this trial is that the importance of graft function may be overestimated due to the evaluation of graft function for 90 days because of short-term drug influences.

## Trial status

Patient enrollment began in September 2016 and is still ongoing. The study is expected to conclude in March 2018. Final results from this RCT are expected in the second quarter of 2018. The results will be published in national and international peer-reviewed journals.

## Additional file

Additional file 1: SPIRIT 2013 Checklist: Recommended items to address in a clinical trial protocol and related documents*. (DOCX 52 kb)

## Abbreviations

IL: interleukin
mTOR: mammalian target of rapamycin
RCT: randomized controlled trial
UTI: urinary tract infection
